# C-851-06. Identifying Risk Factors for Readmission Amongst Unhoused Burn Patients

**DOI:** 10.1093/jbcr/irag033.058

**Published:** 2026-04-06

**Authors:** Victoria R Hammond, Sukruthi Yerramreddy, Michael E Egger, Jason W Smith, Matthew Bozeman

**Affiliations:** University of Louisville Burn Center, Louisville, KY; University of Louisville, Louisville, KY; University of Louisville, Louisville, KY; University of Louisville, Louisville, KY; University of Louisville Burn Center, Louisville, KY

## Abstract

**Introduction:**

Previous studies have used the Nationwide Readmissions Database (NRD) to assess readmission risk factors for patients with burn injury. Other single-institution studies have demonstrated that unhoused burn patients have higher readmission rates than housed burn patients. This study seeks to identify risk factors for readmission amongst unhoused burn patients using national data.

**Methods:**

The 2022 Nationwide Readmissions Database was used for this study. Patients with International Classification of Diseases Version 10 (ICD-10) codes for burn or corrosive injury between January 1, 2022, to November 30, 2022, were included in analysis. Readmission was defined as a subsequent encounter within 30 days with a diagnosis of burn. Univariate analysis was performed with chi-squared and Student’s t tests. Multivariable logistic regression was performed with statistically significant variables on univariate analysis to identify risk factors associated with readmission in unhoused patients.

**Results:**

18607 encounters were identified as index admissions, 1130 (6.1%) were unhoused. Previous research has suggested unhoused patients were likely to leave AMA, have longer lengths of stay, and higher rates of mental illness and substance abuse than housed patients. These findings were supported by our data. 8.6% of unhoused patients (n = 97) were readmitted with a diagnosis of burn injury compared to 5.2% (n = 908) of housed patients (*p*<.001). Risk factors for readmission for unhoused patients included a third-degree burn (OR 2.4, [1.4-4.2], *p*<.001), leaving against medical advice (OR 4.1, [2.6-6.6], *p*<.001), burn of the trunk (OR 1.9, [1.2-3.0], *p*<.001), burn of the upper extremity (OR 1.9, [1.2-3.0], *p*<.001), and burn of the lower extremity (OR 1.6, [1.0-2.6], *p*=.004). Despite higher rates of diagnoses such as schizophrenia, bipolar disorder, alcohol abuse, and/or substance abuse in the unhoused population, these factors did not increase risk for readmission.

**Conclusions:**

Unhoused burn patients are at a higher risk of readmission than housed patients. Severity of injury, location of injury, and leaving against medical advice were factors associated with readmission.

**Applicability of Research to Practice:**

Unhoused burn patients have a high rate of readmission. This study helps identify risk factors for future risk mitigation in this patient population.

**Funding for the Study:**

N/A.

